# Effects of Dietary Fish Oil Levels on Growth Performance, Lipid Metabolism, Hepatic Health, Nonspecific Immune Response, and Intestinal Microbial Community of Juvenile Amur Grayling (*Thymallus grubii*)

**DOI:** 10.1155/anu/8587410

**Published:** 2024-11-21

**Authors:** Shaoxia Lu, Chang'an Wang, Yang Liu, Bing Liu, Ying Zhang, Honghe Shi, Gefeng Xu, Shicheng Han, Hongbai Liu

**Affiliations:** ^1^Laboratory of Aquaculture Nutrition and Feed, Chinese Academy of Fishery Sciences, Heilongjiang River Fisheries Research Institute, Harbin 150070, China; ^2^Key Laboratory of Aquatic Animal Diseases and Immune Technology of Heilongjiang Province, Chinese Academy of Fishery Sciences, Heilongjiang River Fisheries Research Institute, Harbin 150070, China; ^3^Water Ecological Environment Monitoring Center, Inner Mongolia Yin Chao Ji Liao Water Supply Co. Ltd., Ulanhot 137400, China

**Keywords:** growth performance, hepatic health, intestinal microbiota, lipid metabolism, non-specific immune response, *Thymallus grubii*

## Abstract

This trial was conducted to assess the effects of different levels of dietary fish oil on growth performance, hepatic health, nonspecific immune responses, and intestinal microbial community of Amur grayling (*Thymallus grubii*). Five isonitrogenous diets containing 60 (6FO), 90 (9FO), 120 (12FO), 150 (15FO), and 180 g/kg (18FO) fish oil were fed to triplicate groups of 60 fish per tank for 8 weeks, respectively. The results revealed that specific growth rate (SGR) and weight gain (WG) of fish in the 15FO group were significantly greater than those in the 6FO group (*p* < 0.05). Somatic indices and whole-body lipid levels were positively correlated with increases in dietary fish oil levels. Trypsin and lipase activities in 15FO and 18FO groups were significantly higher than those in the 6FO and 9FO groups (*p* < 0.05). The activities of intestinal catalase (CAT) and liver superoxide dismutase (SOD), CAT, lysozyme (LZM), alkaline phosphatase (AKP), and acid phosphatase (ACP) improved significantly as the dietary lipid content increased to 185.3 g/kg and decreased thereafter (*p* < 0.05). The lipid metabolism-related genes peroxisome proliferator-activated receptor gamma (PPAR*γ*) and carnitine palmitoyltransferase 1A (CPT1A) were significantly downregulated and upregulated (*p* < 0.05), respectively, in the 15FO group. Immune-related genes in the liver and intestine, such as interleukin (IL-8), were significantly upregulated in the 15FO group (*p* < 0.05). The liver sections from 18FO group presented more numerous and larger lipid vacuoles. Both low- (6FO) and high-lipid (18FO) diets reduced the relative abundance of intestinal *Lactococcus*. The relative abundances of intestinal *Staphylococcus* and *Bacillus* (mainly *Bacillus anthracis*) increased in the low-lipid diet group and that of *Pedobacter* increased in the high-lipid diet group. Second-order polynomial analysis of WG and the feed conversion ratio (FCR) for varying levels of dietary lipid revealed that a range of 194.76–198.90 g/kg dietary lipid was optimal for the growth and health of Amur grayling.

## 1. Introduction

Lipids are regarded as the main energy source for fish and play a vital role in regulating growth, metabolism, and health [1–3]. The lipid requirements of fish vary among different fish species, life stages, and culture environments [1, 4–8]. Appropriate dietary lipid levels may promote growth, immune, and liver and intestinal health, and also conducive to protein sparing and reduce nitrogen emissions [4, 7, 9–13]. However, excessive lipids may cause liver lipid deposition, inflammatory response, apoptosis, and imbalance of intestinal flora, which can lead to slow growth, increased susceptibility to infections, and disease in fish or death [11, 12, 14–18]. Therefore, determining the quantitative dietary lipid requirements of fish could prevent dietary protein from being utilized as energy, as well as the harmful effects induced by excessive lipid intake.

*Thymallus grubii* is a small and economically cold freshwater fish with the common name “Amur grayling” and is subordinate to Salmoniformes, Salmonidae, *Thymallus*, which is found mainly in the Amur River Basin, China [19]. The fish is delicious and nutritious and has become a popular aquatic product. However, the size of the wild Amur grayling population is declining due to overfishing, pollution, and habitat destruction [19]. To restore its dwindling populations and maintain its biodiversity, juvenile Amur graylings have been stocked annually into the Amur River Basin. In recent years, new advances have been made in the nutritional quality, feeding habit, germplasm identification, artificial production, and rearing of Amur grayling [20], but the nutritional requirements of Amur grayling is investigated little. Fan et al. [20] revealed that dietary 180 g/kg lipids (fish oil:soybean oil = 1:1) could enhance the growth performance, digestive function, and liver antioxidative capacity of juvenile Amur grayling (4.64 ± 0.03 g). However, the fish fed with fish oil and soybean oil as the lipid source showed a low weight gain rate (WGR) and high feed conversion ratio (FCR), which may be due to the mixture lipids could not meet the requirement of juvenile Amur grayling. Thus, the aim of this study was to evaluate the effects of dietary fish oil levels on growth performance, lipid metabolism, hepatic health, nonspecific immune responses, and intestinal microbial community of Amur grayling. The current study is to gain insight into the lipid requirements and serve a basis to improve and optimize the dietary formulations, contributing to the protection, exploitation, and utilization of Amur grayling.

## 2. Materials and Methods

### 2.1. Diet Preparation

Five isonitrogenous (475.74 g/kg crude protein) experimental diets with different dietary fish oil levels were formulated in this study (Table 1). The fatty acid composition of five diets were shown in Table 2. All the solid ingredients were passed through a 60-mesh sieve, weighed, and well mixed. Fish oil and water were subsequently added, and the mixture was thoroughly mixed again. In accordance with standard pelletizing procedures, the dough was extruded into a 1.0 mm diameter pellet with a ring die pellet mill (HKJ-218, Tongli Grain Machinery, China), dried to approximately 10% moisture in a ventilated oven (50 KG, Ketianda Machinery, China) at 60°C, and then stored at −20°C until being used [21].

### 2.2. Fish and Feeding Trial

The Amur graylings used in this experiment were obtained from a farm in Fangzheng County, Harbin Municipality, Heilongjiang Province, Northeast China. After two weeks of acclimation, 900 fish of similar size (1.63 ± 0.06 g) were randomly divided into 15 stainless steel tanks (60 individuals per tank). The feeding trial was conducted in a recirculating aquaculture system (RAS), which comprised 48 stainless-steel tanks (diameter of 0.78 m and height of 0.65 m) and three polypropylene tanks (diameter of 1.9 m and height of 0.80 m). In addition, the water flowed from the rearing tanks and then passed through a water-treatment loop for filtration, removal of ammonia, oxygenation, sterilization, and antisepsis, and eventually flowed back into the rearing tanks to be used again. Approximately 15%–20% of the water was exchanged every day. During the feeding experiment, the fish were hand-fed twice daily (at 8:30 a.m. and at 16:00 p.m.) to apparent satiation for 8 weeks. The trial was conducted under a 12:12-h light:dark photoperiod. To ensure the stability of the water quality, the parameters were monitored every day and maintained at a temperature of 13 ± 0.5°C, a dissolved oxygen concentration of 8.5 ± 0.5 mg/L, a pH of 7.3 ± 7.5, and an ammonia nitrogen content of 0.1 ± 0.02 mg/L.

### 2.3. Sample Collection

After 56 days of the feeding trial, all the fish from each tank were captured, counted, and weighed for further analysis of growth performance. Nine randomly captured fish from each tank were anesthetized in a solution containing 100 mg/L tricaine methanesulfonate (MS-222, Sigma Aldrich, USA). The viscera and liver (three fish per tank) were then excised and weighed to calculate the viscerosomatic index (VSI) and hepatosomatic index (HSI). The gut contents and intestines from nine fish per tank and six liver samples were subsequently collected and immediately stored in liquid nitrogen for further analysis. Another three liver samples were soaked in a solution containing 10% neutral formalin (RightTech, Changchun, China) for histological analysis.

### 2.4. Biochemical Analysis

#### 2.4.1. Proximate Composition Analysis

The proximate composition included moisture content, crude protein, crude lipid, ash, and gross energy. The moisture content was assessed by drying at 105°C in a hot-air oven [22]. The protein content (*N* × 6.25) was measured via a rapid *N* exceed analyzer (Elementar, Germany) [23]. The crude lipid content was determined using Soxhlet extraction, and ash was analyzed using a muffle furnace by incinerating the sample for 4 h at 550°C [21]. The gross energy was determined via an adiabatic bomb calorimeter (IKA C5003, Germany). The gas chromatography system (Agilent, USA) was used to quantify the fatty acid composition of the diets [23].

#### 2.4.2. Hepatic and Intestinal Enzyme Activity Assays

The intestinal and liver samples from three fish per tank were weighed and homogenized with cold 0.9% sodium chloride (NaCl) solution and then centrifuged (Cence, China) at 2500 rpm for 10 min at 4°C to obtain the supernatant for further assessment of digestive enzyme and antioxidant enzyme activity. The activities of intestinal trypsin, lipase, and amylase; intestinal and liver superoxide dismutase (SOD), catalase (CAT), and malondialdehyde (MDA); and liver lysozyme (LZM), alkaline phosphatase (AKP), and acid phosphatase (ACP) were measured using commercial kits (Nanjing Jiancheng Bioengineering Institute, China).

#### 2.4.3. Histological Analysis

The fixed livers were transferred for dehydration in an alcohol series (70%–100%) and embedded in paraffin. The paraffin blocks were sliced to a thickness of 0.5 mm via a microtome, stained with hematoxylin and eosin (H&E), and then sealed with neutral balsam. The sections were examined and documented via a microscopic imaging analyzer (Olympus, Japan).

### 2.5. Gene Expression Analysis

Total RNA was extracted from fresh intestine and liver tissues with TRIzol reagent (Invitrogen, Carlsbad, CA, USA), and complementary DNA (cDNA) was synthesized using kits (Takara, China) in accordance with the manufacturer's instructions. Before cDNA synthesis, total RNA was subjected to electrophoresis on 1.2% (w/v) denatured agarose gel to evaluate its integrity, after which the concentration and 260/280 nm absorbance ratio (close to 2.0) were used to evaluate the quality of the total RNA with a NanoDrop ND-1000 instrument (NanoDrop Technologies, USA). Gene expression levels were determined by ABI 7500 instrument (Applied Biosystems, USA) using a one-step TB Green PrimeScript reverse transcription polymerase chain reaction (RT-PCR) kit (Perfect Real Time). The messenger RNA (mRNA) expression of each sample (*n* = 6) was determined in triplicate and corrected based on *β*-actin, and the fold changes were then evaluated and normalized to the level of the control group via the 2^−*ΔΔCt*^ method. Specific primers for peroxisome proliferator-activated receptor gamma (PPAR*γ*), fatty acid synthase (FAS), carnitine palmitoyltransferase 1A (CPT1A), transforming growth factor beta (TGF-*β*), nuclear factor-kappa B (NF-*κ*B), and interleukin (IL)-8 were designed using the online tool Primer 3 plus (https://www.primer3plus.com/) on the basis of the Amur grayling transcriptome sequence (PRJNA907151), and the application efficiencies (Supporting Information 1) were evaluated according to previously reported methods [24]. The sequences of the primers used for *β*-actin have been previously reported [25]. All primer sequences used in this study are shown in Supporting Information 1.

### 2.6. Intestinal Microbiota Analysis

The gut contents from three fish were mixed into one sample. The extraction of intestinal microbial (*n* = 3) total DNA was conducted using a commercial DNA extraction kit (MP Biomedicals, Irvine, CA, USA), and the integrity and quality of total DNA were also evaluated via agarose gel electrophoresis and microvolume ultraviolet (UV)–visible (Vis) spectrophotometry. Subsequently, the V3–V4 hypervariable regions of the bacterial 16S rRNA were amplified and sequenced on an Illumina MiSeq platform (Illumina, San Diego, CA, USA) to construct a library.

For bioinformatic analysis of intestinal bacteria, the 16S rRNA amplicon sequences were clustered into operational taxonomic units (OTUs) on the basis of sequence similarity (97%) via the QIIME pipeline (version 1.8.0)[26]. The alpha diversity and beta diversity analyses were performed according to Liu's method [21]. Venn diagrams were used to analyze the unique and shared OTUs between groups. The differences in intestinal bacteria between groups were identified at the phylum and genus levels. Linear discriminant analysis effect size (LEfSe) was conducted to identify genera with differential abundances between groups fed diets with different concentrations of fish oil. Differences between populations were analyzed via the Kruskal–Wallis test. *p* < 0.05 was considered to indicate statistical significance.

### 2.7. Statistical Analysis

One-way analysis of variance (ANOVA) and Duncan's multiple range test were carried out with Statistical Package for the Social Sciences (SPSS) 22.0 statistical software (IBM, USA) to observe significant differences (*p* < 0.05) among the five groups. A second-order polynomial regression model was performed using OriginPro 2021 software to determine the optimum lipid level.

## 3. Results

### 3.1. Growth Performance, Feed Utilization, and Body Indices

**Figure 1:**
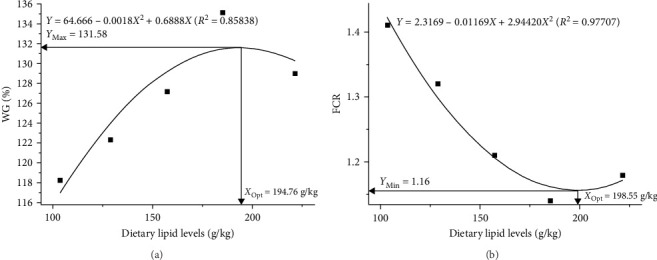
Quadratic regression analysis of the (a) WG and (b) FCR against varying levels of dietary fish oil. FCR, feed conversion ratio; Opt, optimal; WG, weight gain.

As shown in Table 3, the final body weight (FBW) increased with increasing dietary fish oil level, and the FBW of fish fed diets with 150 g/kg (15FO) and 180 g/kg (18FO) fish oil was significantly greater than that of fish fed diets with 60 (6FO)–120 g/kg (12FO) fish oil (*p* < 0.05). The protein efficiency ratio (PER), WGR, and specific growth rate (SGR) first increased but then decreased with increasing dietary fish oil level. The values of these parameters in the 15FO group were significantly greater than those in the 6FO group (*p* < 0.05). The FCR first decreased but then increased with increasing dietary fish oil level up to 180 g/kg, and the FCR of the 15FO and 18FO groups was significantly lower than that of the 6FO and 9 g/kg fish oil (9FO) groups (*p* < 0.05). The viscerosomatic index (VSI) and survival rate of the 18FO group were significantly greater than those of the other groups (*p* < 0.05). However, the condition factor (CF) and hepatosomatic index (HSI) of Amur grayling did not significantly differ among the groups (*p* > 0.05). Quadratic analyses of weight gain (WG) and FCR indicated that the optimal lipid requirements for Amur grayling were between 194.76 and 198.90 g/kg diet (Figure 1).

### 3.2. Proximate Composition of Fish

The data from the proximate composition analysis are presented in Table 4. Different dietary fish oil levels significantly influenced the moisture, crude protein, and crude lipid contents of Amur grayling (*p* < 0.05). A decreasing trend in whole-body moisture, crude protein, and ash and an increasing trend in crude lipid content were observed with increasing dietary fish oil levels. These parameters did not vary significantly among the fish in the 6FO, 9FO, and 12FO groups (*p* > 0.05), but the values found for these groups were significantly different from those obtained for the 15FO and 18FO groups (*p* < 0.05). However, dietary fish oil levels had no significant influence on the ash content of the fish (*p* > 0.05).

### 3.3. Enzyme Activity in the Intestine and Liver

The effects of fish oil levels on intestinal and liver enzyme activities in Amur grayling are shown in Table 5. A positive correlation was found between the activities of intestinal trypsin and lipase and dietary fish oil levels. The fish fed diets with 150 and 180 g/kg fish oil presented significantly increased intestinal lipase activities compared with those fed diets with 60–90 g/kg fish oil (*p* < 0.05), and significantly higher trypsin activities were found in the fish fed 150 and 180 g/kg fish oil than in the other groups (*p* < 0.05). However, there were no significant differences in intestinal *α*-amylase among the groups (*p* > 0.05). The intestinal SOD activity of the 15FO and 18FO groups was significantly greater than that of the other groups (*p* < 0.05). The intestinal CAT activities were significantly greater in the 15FO group than in the groups fed with 60–120 g/kg fish oil (*p* < 0.05). Moreover, different dietary fish oil levels significantly influenced the liver SOD and CAT activities of Amur grayling (*p* < 0.05). These parameters did not vary significantly among the fish in the 6FO and 9FO groups (*p* > 0.05), but the values found for these groups were significantly different from those obtained for the 15FO and 18FO groups (*p* < 0.05). The intestinal and liver MDA activities significantly decreased with increasing dietary fish oil levels up to 150 g/kg and then significantly increased with further increases in dietary fish oil levels up to 180 g/kg (*p* < 0.05). The activities of liver LZM, AKP, and ACP in Amur grayling significantly increased with increasing levels of dietary fish oil levels up to 150 g/kg and then significantly decreased with further increases in the levels of dietary fish oil up to 180 g/kg (*p* < 0.05).

### 3.4. Expression of Lipid Metabolism-Related and Immune-Related Genes

**Figure 2:**
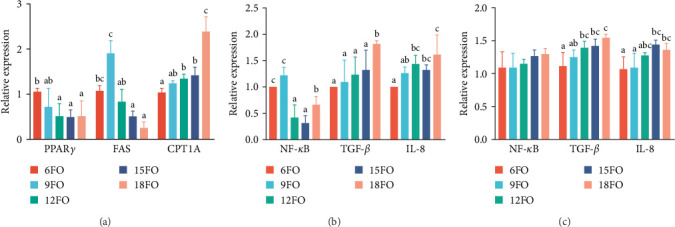
Effects of dietary fish oil levels on the relative mRNA expression of liver lipid metabolism-related genes (PPAR*γ*, FAS, and CPT1A) (a), Liver immune–related genes (NF-*κ*B, TGF-*β*. and IL-8) (b), and intestinal immune–related genes (NF-*κ*B, TGF-*β*, and IL-8) (c). Results are expressed as means ± S.E (*n* = 6), and different letters above a bar denote the significant difference between treatments (*p* < 0.05). CPT1A, carnitine palmitoyltransferase 1A; FAS, atty acid synthase; IL, interleukin; mRNA, messenger RNA; PPAR*γ*, peroxisome proliferator-activated receptor gamma; NF-*κ*B, nuclear factor-kappa B; TGF-*β*, transforming growth factor beta.

The liver lipid metabolism-related gene expression of Amur grayling is presented in Figure 2a. At dietary fish oil levels greater than 120 g/kg, the mRNA expression level of PPAR*γ* was significantly lower than that in the 6FO group (*p* < 0.05). The mRNA expression of FAS in the 9FO group was greater than that in the 6FO group and was significantly greater than that in the groups fed diets with fish oil levels greater than 120 g/kg (*p* < 0.05). CPT1A mRNA expression increased with increasing fish oil content and was significantly upregulated in the 18FO group compared with the other groups (*p* < 0.05).

Figure 2b shows the expression of immune-related genes in the liver. With increasing dietary fish oil levels, the mRNA expression of NF-*κ*B initially increased in the 9FO group, then significantly decreased in the 12FO and 15FO groups, and subsequently significantly increased in the 18FO group (*p* < 0.05). The mRNA expression levels of TGF-*β* were significantly greater in the 18FO group than in the other groups (*p* < 0.05). The mRNA expression of IL-8 was significantly greater in the 18FO group than in the 6FO and 9FO groups, and the levels in the 12FO and 15FO groups were significantly greater than those in the 6FO group (*p* < 0.05).

Figure 2c shows the expression of immune-related genes in the intestine. Dietary fish oil levels had no significant influence on the mRNA expression of NF-*κ*B (*p* > 0.05). The mRNA expression of TGF-*β* and IL-8 increased with increasing dietary fish oil levels and was significantly greater in the 15FO and 18FO groups than in the 6FO group (*p* < 0.05).

### 3.5. Liver Histology

**Figure 3:**
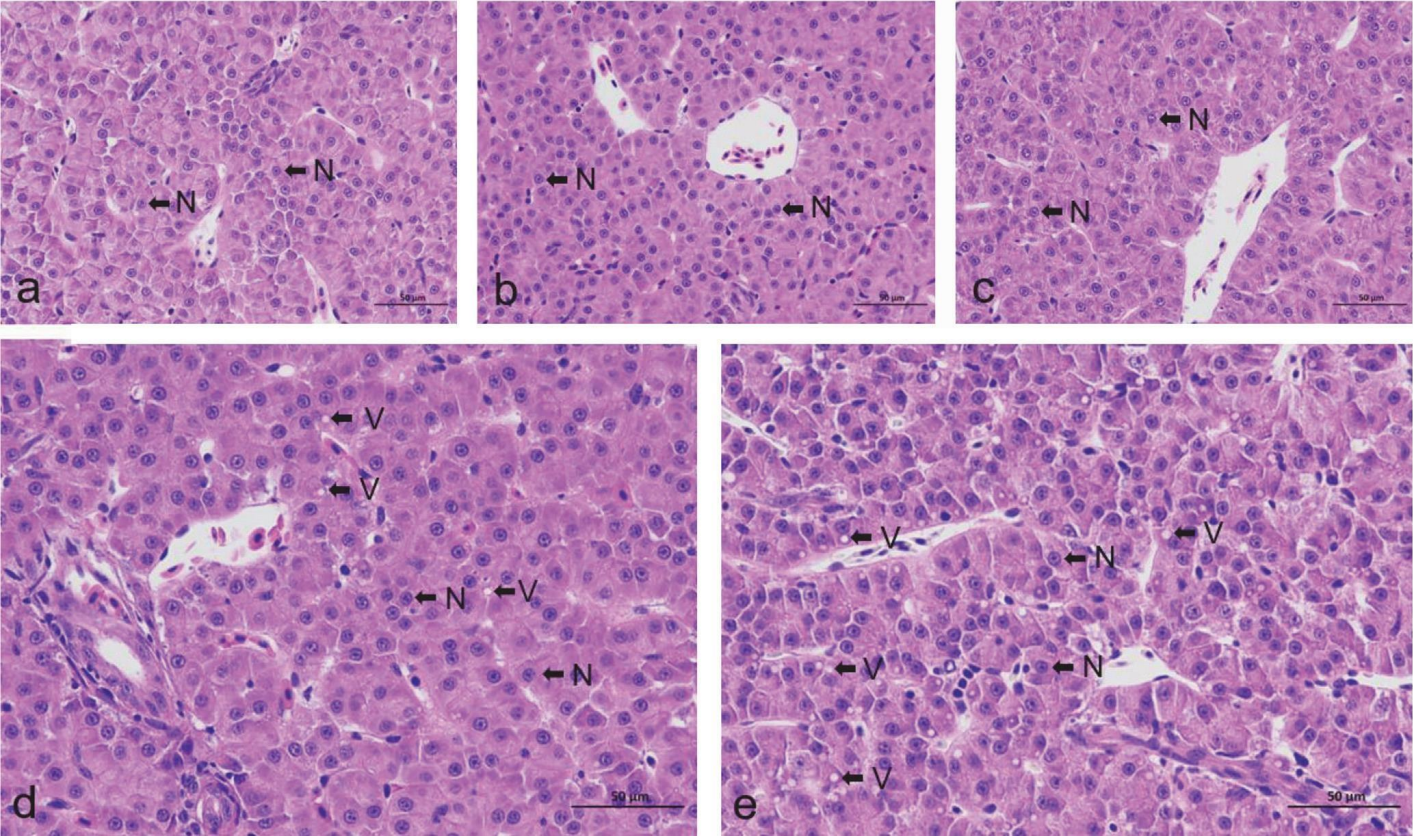
Histological examination of the livers of Amur grayling fed different fish oil levels. (a) 6FO, (b) 9FO, (c) 12FO, (d) 15FO, and (e) 18FO (H&E). 6FO, 60 g/kg fish oil; 9FO, 90 g/kg fish oil; 12FO, 120 g/kg fish oil; 15FO, 150 g/kg fish oil; 18FO, 180 g/kg fish oil; H&E, hematoxylin and eosin; N, hepatocyte nuclei; V, lipid vacuoles.

As shown in Figure 3, the liver structure of the fish fed a diet containing 60–120 g/kg fish oil was complete. The liver cells were tightly arranged radially around the central vein, the cell boundaries were clear, and most of the nuclei were in the center of the cell (Figure 3a–c). In the fish fed 150 and 180 g/kg fish oil, the nuclei were gradually shifted towards one side, white transparent lipid vacuoles appeared, and their abundance and size were affected by the dietary fish oil levels (Figure 3d,e).

### 3.6. Intestinal Microbiota

In total, 7,30,531 high-quality sequences (average length of 426 bp) were collected from 15 samples (three per group). All the raw sequencing data were submitted to National Center for Biotechnology Information (PRJNA1043406). At the end of the rarefaction curves, the data tended to saturate, indicating that the sequencing depth of the raw data was sufficient and could be used for further analysis (Supporting Information 2). As shown in Supporting Information 3, significant differences in alpha diversity, including the Shannon, Simpson, Abundance-based Coverage Estimator (ACE), Chao1, and coverage indices, were not found among the treatment groups (*p* > 0.05). Analysis of the beta diversity at the genus level showed that differences between groups were found only in the binary Jaccard Principal Coordinate Analysis (PCoA) plot (*p* < 0.05; Supporting Information 4).

**Figure 4:**
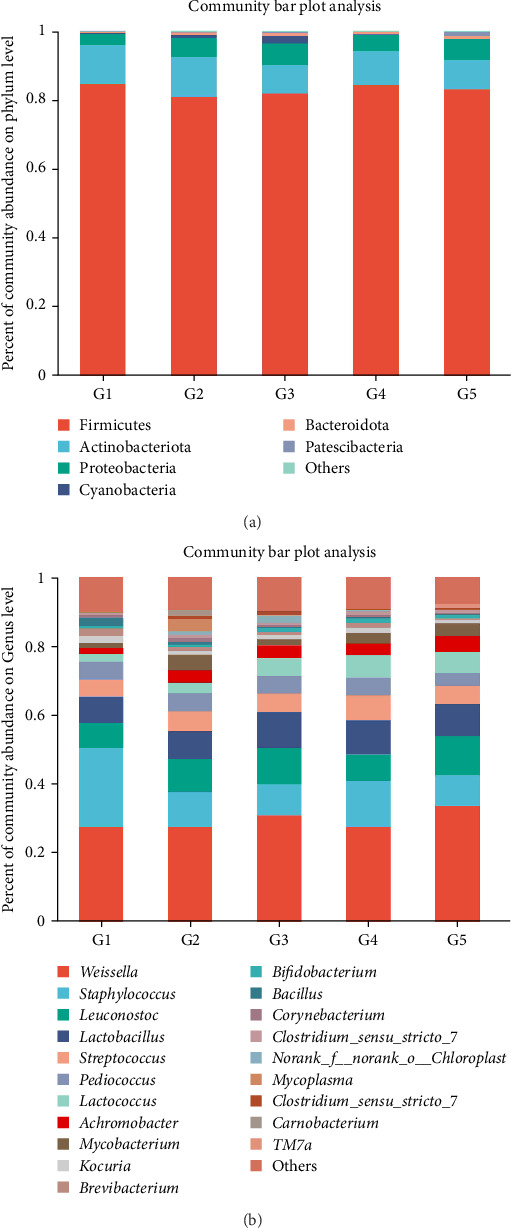
Effects of dietary lipid levels on the relative abundance of intestinal microbes in juvenile Amur grayling at the phylum (a) and genus (b) levels. G1 : 6FO, G2 : 9FO, G3 : 12FO, G4 : 15FO and G5 : 18FO. 6FO, 60 g/kg fish oil; 9FO, 90 g/kg fish oil; 12FO, 120 g/kg fish oil; 15FO, 150 g/kg fish oil; 18FO, 180 g/kg fish oil.

**Figure 5:**
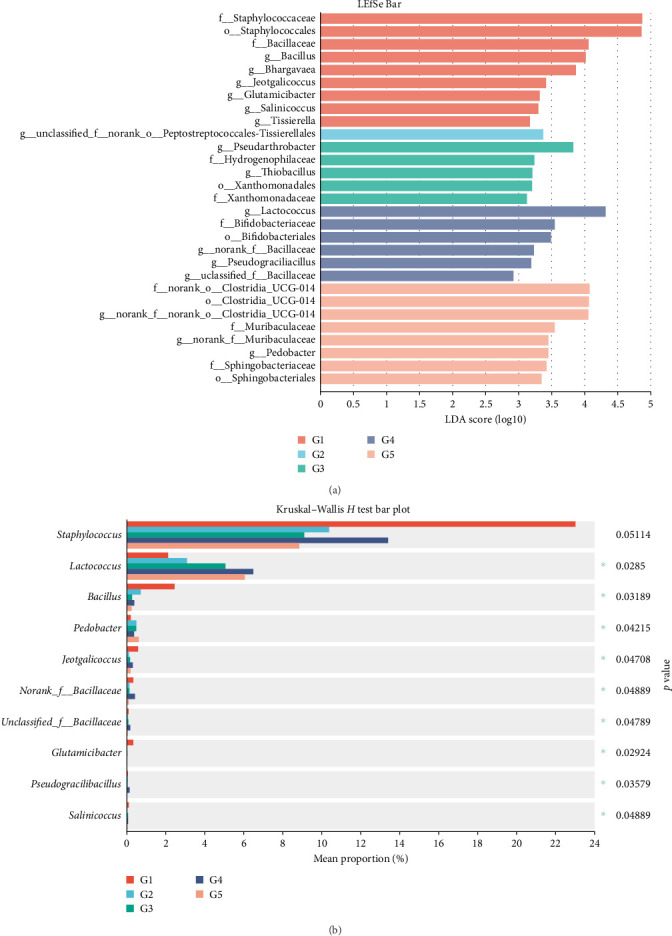
LEfSe and Kruskal–Wallis *H* test analysis of the differential abundance of taxa within Amur grayling intestinal microbiota following random sampling from each group. (a) LDA score of the abundance of different taxa. (b) Kruskal–Wallis *H* test bar graph for special microbial composition at genus levels. G1 : 6FO, G2 : 9FO, G3 : 12FO, G4 : 15FO and G5 : 18FO. LEfSe at different levels of each group. The length of the histogram represents the abundance of the difference species. 6FO, 60 g/kg fish oil; 9FO, 90 g/kg fish oil; 12FO, 120 g/kg fish oil; 15FO, 150 g/kg fish oil; 18FO, 180 g/kg fish oil; LDA, linear discriminant analysis; LEfSe, linear discriminant analysis effect size.

**Figure 6:**
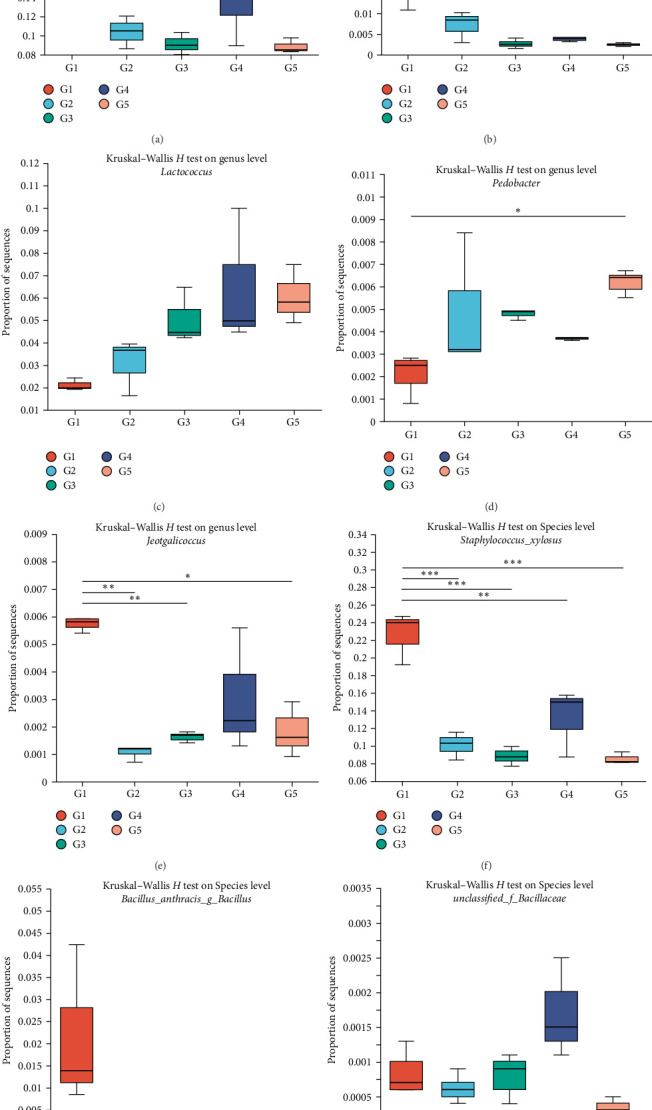
Kruskal–Wallis *H* test analysis the significance of differences of the relative abundances of some genera and species between groups. (a) *Staphylococcus*, (b) *Bacillus*, (c) *Lactococcus*, (d) *Pedobacter*, (e) *Jeotgalicoccus*, (f) *Staphylococcus xylosus*, (g) *Bacillus anthracis*, and (h) *unclassified_f_Bacillaceae*. G1 : 6FO, G2 : 9FO, G3 : 12FO, G4 : 15FO and G5 : 18FO. The identical indexes with ⁣^*∗*^,  ^*∗*^^*∗*^, and  ^*∗*^^*∗*^ had obviously different mean values (*p* < 0.05, *p* < 0.01, and *p* < 0.001, respectively). 6FO, 60 g/kg fish oil; 9FO, 90 g/kg fish oil; 12FO, 120 g/kg fish oil; 15FO, 150 g/kg fish oil; 18FO, 180 g/kg fish oil.

In total, 169 shared OTUs between groups were identified via Venn diagram analysis, and the number of OTUs first increased but then decreased as the level of dietary fish oil increased (Supporting Information 5). The relative abundances of the phyla and genera accounting for more than 1% of the gut microbiota are shown as stacked histograms (Figure 4a,b). As illustrated in Figure 4a, the dominant phyla in all the groups were Firmicutes and Actinobacteria. At the genus level, the greatest difference between groups in the relative abundance of any genus was found for *Staphylococcus* between the 6FO group and the other groups (Figure 4b). LEfSe analysis revealed a total of 29 taxa with significant differences among the groups (Figure 5a). The fish fed diets containing 150 g/kg fish oil presented significantly increased relative abundances of Bacillaceae (*g_norank_f_Bacillaceae* and *g*_unclasfied_f_Bacillaceae) and *Lactococcus* (linear discriminant analysis (LDA) score >2 and *p* < 0.05). The results of the Kruskal–Wallis *H* test revealed significant differences among groups in the abundances of *Lactococcus*, *Bacillus*, *Pedobacter*, *Jeotgalicoccus*, *norank_f_Bacillaceae*, *unclassified_f_Bacillaceae*, and 19 other genera (*p* < 0.05; Figure 5b). The Kruskal–Wallis *H* test was conducted to further analyze the differences in several special genera, including *Lactococcus*, *Bacillus*, *Staphylococcus*, and *B. anthracis*, between the groups, revealing that the relative abundances of *Staphylococcus* and *Staphylococcus xylosus* in the 6FO group were significantly greater than those in the other groups (*p* < 0.05; Figure 6a–c,f). The relative abundances of *Bacillus* and *Bacillus anthracis* decreased as the fish oil level increased (*p* > 0.05; Figure 6b,g); in addition, the relative abundance of *unclassified_f_Bacillaceae* first increased and then decreased as the fish oil level increased, and its abundance in the 15FO group was significantly greater than that in the 18FO group (Figure 6h; *p* < 0.05). The relative abundance of *Pedobacter* first increased, then decreased in the 15FO group, and subsequently increased in the 18FO group, and the relative abundance in the 18FO group was significantly greater than that in the 6FO group (Figure 6d; *p* < 0.05). The relative abundance of *Jeotgalicoccus* first decreased, then increased, and subsequently decreased, and the relative abundance in the 6FO group was significantly greater than that in the other groups except for the 15FO group (Figure 6e; *p* < 0.05).

## 4. Discussion

In the present study, the WG and SGR increased and the FCR decreased with increasing dietary fish oil content to 150 g/kg, and these parameters showed the opposite trend as the dietary fish oil level increased from 150 to 180 g/kg, which indicates that a diet containing 180 g/kg fish oil had no further beneficial effects on Amur grayling. These observations are similar with findings by Fan et al. [20]. Different from study by Fan et al. [20], the FCR and WGR in fish fed with fish oil as a single lipid (FCR: 1.14–1.41 and WGR: 118.20%−135.10%) in our study were much better than that in fish fed with fish oil and soybean oil as the lipid source (FCR: 1.45–1.91 and WGR: 72.61%–123.75%). These differences may be influenced by the mixture diets, which could not meet the requirement of fatty acid for Amur grayling. So, fish oil may be more suitable than mixture (fish oil and soybean oil) as the lipid sources for cultivation of juvenile Amur grayling. Similar observations about these parameters have been obtained with spotted seabass (*Lateolabrax maculatus*), Atlantic halibut (*Hippoglossus hippoglossus*, L.), chu's croaker (*Nibea coibor*), and golden pompano (*Trachinotus ovatus*) with increasing dietary lipid levels [27–30]. This change may occur because excessive lipid levels suppress de novo fatty acid synthesis and decrease digestion and assimilation in fish [31]. The protein-sparing effect of lipid reflect on the PER and growth. In our study, the PER increased with increasing dietary fish oil levels up to 150 g/kg and then decreased, suggesting that an appropriate fish oil levels enhances a higher protein-sparing effect and feed efficiency, and also decreased unnecessary lipid deposition in the peritoneal cavity and liver. Considering the feed cost, feed efficiency, and the balance between growth performance and economic, dietary fish oil level should be around 150 g/kg (185.3 g/kg lipids of feed). Quadratic analyses of WG and FCR revealed that the optimal lipid requirements for juvenile Amur grayling range from 194.76 to 198.90 g/kg diet. This level is lower than that reported for brown trout (*Salmo trutta*; 290 g/kg), Atlantic salmon (*Salmo salar*; 380–470 g/kg), and rainbow trout (>260.6 g/kg) in cage culture with flowing water [7, 32, 33]. These differences are influenced by a variety of factors, such as life stage, cultivation environment, and feed formula [33, 34]. The dietary fish oil level significantly affects the VSI of Amur grayling. Similar results have been reported in rainbow trout [34]. This finding indicates that excess dietary lipids lead to unnecessary lipid deposition in the peritoneal cavity and liver.

As a vital indicator of the nutrition and quality of fish, the body composition of fish is usually altered by dietary lipid levels. In the present study, a significant decrease in whole-body moisture and crude protein and an increase in crude lipid content were observed as the dietary fish oil levels increased to 150 g/kg, and no significant difference in body ash content was found, which may be due to fat deposition in the body. This finding is consistent with the results obtained for spotted knifejaw (*Oplegnathus punctatus*) and Senegalese sole (*Solea senegalensis*) [35, 36].

The activities of digestive enzymes affect the nutrient digestion and absorption abilities of fish and thus, affect their growth rate and development [37, 38]. In the present study, no significant differences in amylase activity were found among the dietary treatments [1]. We speculate that this finding might be due to the poor ability of carnivorous fish to utilize carbohydrates, which is consistent with the results obtained with rainbow trout [39]. However, the activity of intestinal lipase increased as the dietary fish oil content increased, indicating that Amur grayling might increase lipase activity to improve the digestion and absorption of dietary lipids to adapt to increases in dietary fish oil levels. Similar results have been reported for rainbow trout, brown trout, and lumpfish (*Cyclopterus lumpus*) [40–42]. The activity of trypsin also increased as the levels of dietary fish oil increased, which was similar to previous findings in lumpfish [40]. The changes in protease and lipase activity observed in our study differed from the trend of increasing and then declining with increases in the dietary lipid levels found in turbot and orange-spotted grouper larvae (*Epinephelus coioides* H.) [6, 43]. This difference may arise from differences in dietary lipid levels and species.

Generally, excessive dietary lipids tend to cause lipid peroxidation [1, 12]. As vital responsive elements of the antioxidative defense system, CAT and SOD are involved in scavenging reactive oxygen species (ROS) and thus, maintaining immune homeostasis and protecting cells from free radical damage [44, 45]. In this study, the liver SOD and CAT activities and intestinal CAT activity increased with increasing dietary fish oil levels up to 150 g/kg and then decreased with further increase in dietary fish oil, which is in agreement with the results obtained in Amur grayling fed with mixture lipids [20]. Moreover, the liver and intestinal MDA activities first decreased and then increased as the lipid level increased, which is similar to the findings obtained for rainbow trout and turbot [7, 43]. This observation suggests that an optimal lipid level is beneficial for increasing antioxidant activity and that excess dietary lipids induce lipid peroxidation in Amur grayling.

Innate immunity is a vital defense against foreign pathogen invasion. LZM, AKP, and ACP are important components of the nonspecific immune response and are involved in the killing or digestion of pathogens [46, 47]. Previous studies have shown that oxidative stress caused by increased lipid accumulation in fish fed a high-energy diet can induce an inflammatory response [14, 48–51]. In the present study, the activities of LZM, AKP, and ACP in the liver significantly increased with increasing dietary fish oil levels up to 150 g/kg and then decreased, which is similar to the findings obtained in turbot [43]. These observations indicate that an optimal lipid level is beneficial for intensifying nonspecific immunity and that excessive lipid levels may trigger liver inflammation in Amur grayling.

The expression of genes related to lipid metabolism was analyzed to further elucidate the effects of different lipid levels on the regulation of liver lipid metabolism in Amur grayling. FAS plays a crucial role in the de novo synthesis of fatty acids [52]. PPAR*γ* (PPARG) is a key transcription regulator that regulates lipid metabolism and storage to maintain liver lipid homeostasis [53]. The present study revealed that the relative expression of CPT1A increased as the dietary fish oil levels increased, indicating that high levels of dietary fish oil increase lipolysis in Amur grayling, which is similar to the findings obtained in Japanese seabass fed diets containing fish oil and plant oil [14]. The expression of PPAR*γ* and FAS decreased as the dietary fish oil levels increased, suggesting that endogenous lipid synthesis in the liver decreases with increasing dietary fish oil levels. These observations were consistent with the results reported for rainbow trout, turbot (*Psetta maxima*), and orange-spotted grouper larvae [6, 15, 54]. Over all, the fatty acids composition of 15FO could meet the requirement of Amur grayling.

Increasing evidence indicates that liver and intestinal inflammation induced by a high-fat diet is related to Toll-like receptors (TLR) signal transduction in fish [49, 55, 56]. TLR-mediated signals are transduced mainly through myeloid differentiation primary response gene 88 (MyD88), which subsequently results in the activation of the downstream regulator NF-*κ*B and thus, enhances the expression of pro-inflammatory cytokines [57, 58]. NF-*κ*B and IL-8 are pro-inflammatory factors and TGF-*β* is an anti-inflammatory cytokine that controls the negative effects induced by pro-inflammatory cytokines [47]. In this study, the expression of IL-8 and TGF-*β* in Amur grayling increased with increasing dietary fish oil levels, and the expression of NF-*κ*B first increased, then decreased and subsequently increased, suggesting that a high-fat diet activates pro-inflammatory and anti-inflammatory responses in fish, which is similar to the results obtained for Japanese seabass and tilapia (*Oreochromis niloticus*) fed high-fat diets containing fish oil and plant oil [14, 57].

Extensive research has demonstrated that excessive dietary lipid levels result in lipid deposition in the liver and muscles and thus, result in disturbance of lipid metabolism and hepatocyte damage [57, 58]. The present study revealed that in the liver sections of the fish fed the diet with 221.6 g/kg lipids, the nuclei had shifted to one side of cell, and cells contained more numerous and larger vacuoles than those of the fish fed the diet with 60–150 g/kg fish oil, suggesting that dietary excessively fish oil increased the lipids accumulation in the liver of Amur grayling, and thus, resulting in hepatocyte damage and metabolic disorders. Similar results have been reported in giant grouper (*Epinephelus lanceolatus*) and Yangtze sturgeon (*Acipenser dabryanus*) fed with fish oil and soybean oil [58, 59]. Although the fish did not exhibit severe liver injury during this 8-week feeding trial, they were still at risk of severe hepatocyte damage resulting from excessive lipid (221.6 g/kg) intake. A prolonged feeding period may cause health problems or even death in Amur grayling.

The gut microbiota is indispensable for maintaining fish health and is involved in stimulating the immune system, absorbing nutrients, and regulating lipid, glucose, and energy metabolism [43, 60]. In the present study, although no significant differences were found in the alpha diversity index among the compared groups, increases in the dietary fish oil content first increased and then decreased the number of OTUs at the genus level and altered the abundance of the microbiome at the genus level, suggesting that an optimal lipid level is beneficial for maintaining the intestinal microbiota structure and diversity of Amur grayling. Firmicutes and Actinobacteria were identified as the dominant bacterial phyla in the five groups, which was different from the results of previous studies on intestinal microbiota of turbot, hybrid yellow catfish (*Tachysurus fulvidraco*♀ × *Pseudobagrus vachellii*♂), and golden pompano [30, 43, 61]. These observations may be correlated with the species, dietary lipid levels, and breeding environment.

It is well known that some bacteria of the genus *Staphylococcus* can be opportunistic pathogens [62]. For example, *S. xylosus* has been proven to lead to exophthalmia, break the primary immune barrier, and even cause mortality in rainbow trout [63]. *Bacillus* species, including *Bacillus coagulans*, *Bacillus amyloliquefaciens*, and *Bacillus subtilis*, are applied in aquaculture to improve growth performance and immune function and inhibit the growth of pathogens [64, 65]. However, *B. anthracis* is a well-known pathogen that infects the skin, lungs, or intestines, resulting in severe damage to tissues and organs [66]. In our study, the relative abundances of *Staphylococcus* (mainly *S. xylosus*) and *Bacillus* (mainly *B. anthracis*) decreased when the dietary fish oil level was greater than 60 g/kg, indicating that dietary lipids can reduce harmful bacteria to maintain intestinal health. *Lactococcus* is usually considered a probiotic that promotes growth performance, improves disease resistance, and enhances immune responses in fish [67, 68]. In the present study, LEfSe analysis revealed that feeding fish a diet containing 150 g/kg fish oil significantly increased the relative abundances of Bacillaceae (*g_norank_f_Bacillaceae* and *g_unclassified_f_Bacillaceae*) and Lactococcus, suggesting that an optimal lipid level is beneficial for promoting the proliferation of beneficial intestinal bacteria. *Pedobacter* belongs to Sphingobacteriaceae and Bacteroidetes, which are widely distributed in the natural environment. *Pedobacter* is a pathogenic bacterium in giant pandas and has been detected in the gills of Atlantic salmon prior to gill disease [69, 70]. Although the function of *Pedobacter* in fish remains uncharacterized, in our study, the relative abundance of *Pedobacter* in the 18FO group was significantly greater than that in the other groups. *Jeotgalicoccus* has been isolated from the soft tissues of *Manila clams* via mercury (Hg) bioaccumulation [71]. In broiler chickens, *Jeotgalicoccus* is negatively correlated with indole, choline, taurodeoxycholic acid, and dehydrocholic acid, indicating that it is negatively associated with the synthesis of bile acid [72]. Although the function of *Jeotgalicoccus* in fish is currently uncharacterized, in our study, the relative abundance of *Jeotgalicoccus* in the 6FO group was significantly greater than that in the other groups, possibly associated with the lower secretion of bile acid in the low-lipid diet group.

## 5. Conclusion

In conclusion, fish fed with fish oil as a single lipid source may be suitable for juvenile Amur grayling to enhance the growth and feed efficiency compared with fish fed fish oil and soybean oil. High dietary fish oil levels depress nonspecific immunity, induce lipid peroxidation and potential hepatic lesions and affect the composition and diversity of the intestinal microbiota in Amur grayling. Diets containing 194.76–198.90 g/kg lipids were optimal for growth, hepatic health, nonspecific immune responses, and intestinal microbial community of juvenile Amur grayling. Those data would provide basis for improving and optimizing the dietary formulations to promote a sustainable culture of this fish species.

## Figures and Tables

**Table 1:** Composition of experimental diets.

Diet	6FO	9FO	12FO	15FO	18FO
Fishmeal^a^	400	400	400	400	400
Corn protein concentrate^b^	40	40	40	40	40
Soy protein concentrate^c^	150	150	150	150	150
Wheat gluten meal^d^	85	85	85	85	85
Wheat flour	109.8	109.8	109.8	109.8	109.8
Fish oil^e^	60	90	120	150	180
Vitamin and mineral mix^f^	10	10	10	10	10
Microcrystalline cellulose^g^	120	90	60	30	0
Methyl cellulose^h^	10	10	10	10	10
Sodium alginate	10	10	10	10	10
Chloride choline^i^	5	5	5	5	5
BHT^j^	0.2	0.2	0.2	0.2	0.2
Total	1000	1000	1000	1000	1000
Approximate chemical composition (% of dry matter)					
Moisture	4.51	5.45	5.60	5.13	4.35
Crude protein	47.66	47.58	47.51	47.65	47.47
Crude lipid	10.35	12.89	15.73	18.53	22.16
Ash	10.15	10.14	10.16	10.15	10.14
Gross energy (MJ/kg)	17.88	19.08	20.27	21.47	22.66

Abbreviations: 6FO, 60 g/kg fish oil; 9FO, 90 g/kg fish oil; 12FO, 120 g/kg fish oil; 15FO, 150 g/kg fish oil; 18FO, 180 g/kg fish oil; BHT, butylated hydroxytoluene.

^a^Fishmeal: from TASA Fish Product Co., Ltd. Peru.

^b^Corn protein concentrate: Henan Julong Biogical Engineering Co., Ltd., Henan, China.

^c^Soy protein concentrate: Yihai (Taizhou) Grain and Oil Industry Co., Ltd., Jiangsu, China.

^d^Wheat gluten meal: Henan Midaner Trading Co., Ltd., Henan, China.

^e^Fish oil: from Foshan city Damao Feed Co., Ltd., Guangdong, China.

^f^Vitamin and mineral mix (mg/kg or IU/kg diet): VA: 750,000 IU, VD3: 200,000 IU, VE: 6000 mg, VK3: 2000 mg, VB1: 1,200 mg, VB2: 1200 mg, VB6: 1200 mg, VB12: 8 mg, VC 21000 mg, D-calcium pantothenate: 2000 mg, niacinamide: 9000 mg, folic acid: 370 mg, D-biotin: 15 mg, inositol: 10,000 mg, MgSO_4_: 6000 mg, ZnSO_4_: 4000 mg, MnSO_4_: 2500 mg, CuSO_4_: 2500 mg, FeSO_4_: 2500 mg, CoSO_4_: 160 mg, Ca(IO_3_)_2_: 200 mg, and Na_2_SeO_3_: 40 mg.

^g^Microcrystalline cellulose, >99.0%, Aladdin Biochemical Technology, Co., Ltd., Shanghai, China.

^h^Methyl cellulose, Sigma-aldrich (Shanghai) Trading Co., Ltd., Shanghai, China.

^i^Chloride choline, 50%, Jujia Biotech Co., Ltd., Shandong, China.

^j^BHT, >99.0%, Aladdin Biochemical Technology, Co., Ltd., Shanghai, China.

**Table 2:** Fatty acid profilles (% total fatty acids) of the experimental diets.

Fatty acid	6FO	9FO	12FO	15FO	18FO
C13:0	0.09	0.09	0.09	0.09	0.09
C14:0	4.28	4.03	3.76	3.45	3.30
C15:0	1.04	1.03	0.99	0.99	0.97
C16:0	11.74	10.55	9.40	8.54	7.33
C17:0	1.20	1.19	1.15	1.10	1.02
C18:0	5.28	4.90	4.74	4.50	4.34
C20:0	1.61	1.54	1.49	1.42	1.38
C21:0	0.18	0.20	0.20	0.19	0.20
C22:0	0.63	0.59	0.55	0.48	0.44
C23:0	0.17	0.16	0.15	0.14	0.13
C24:0	0.64	0.55	0.51	0.47	0.43
C16:1	6.33	6.43	6.53	6.40	6.31
C20:1	2.71	2.61	2.53	2.45	2.35
C20:2	0.13	0.19	0.23	0.27	0.21
C24:1	1.38	1.27	1.18	1.12	1.06
C18:1*n*9*c*	21.97	22.68	23.17	23.76	24.75
C22:1*n*9	5.57	6.63	7.62	8.61	9.29
C18:2*n*6*c*	7.69	6.48	5.43	4.60	3.68
C20:4*n*6	4.57	4.31	4.25	4.04	3.76
C18:3*n*3	2.49	2.17	2.00	1.80	1.48
C20:3*n*3	0.29	0.31	0.30	0.28	0.34
C20:5*n*3	10.69	11.13	11.75	12.49	12.98
C22:6*n*3	9.32	10.95	11.99	12.82	14.17
∑SFA	26.87	24.83	23.03	21.37	19.63
∑MUFA	38.08	39.81	41.26	42.61	43.96
∑PUFA	35.05	35.37	35.71	36.01	36.41
∑*n–*3 FA	22.79	24.57	26.04	27.38	28.97
∑*n*–6 FA	12.26	10.79	9.67	8.63	7.44
*n*–3/*n*–6	1.86	2.28	2.69	3.17	3.89
DHA/EPA	0.87	0.98	1.02	10.3	1.09

Abbreviations: 6FO, 60 g/kg fish oil; 9FO, 90 g/kg fish oil; 12FO, 120 g/kg fish oil; 15FO, 150 g/kg fish oil; 18FO, 180 g/kg fish oil. DHA, docosahexaenoic acid; EPA, eicosapentaenoic acid; FA, fatty acid; MUFA, monounsaturated fatty acid; PUFA, polyunsaturated fatty acid; SFA, saturated fatty acid.

**Table 3:** Growth performance, feed utilization, and morphometric parameters of Amur grayling fed different lipid diets.

Item	6FO	9FO	12FO	15FO	18FO
IBW (g)	1.61 ± 0.07	1.62 ± 0.05	1.62 ± 0.08	1.63 ± 0.05	1.69 ± 0.05
FBW (g)	3.55 ± 0.04^a^	3.60 ± 0.03^ab^	3.68 ± 0.07^b^	3.78 ± 0.04^c^	3.87 ± 0.06^c^
WG (%)	118.25 ± 11.68^a^	122.32 ± 8.57^ab^	127.15 ± 5.86^ab^	135.10 ± 6.47^b^	128.99 ± 3.67^ab^
SGR (%/day)	1.39 ± 0.10^a^	1.43 ± 0.07^ab^	1.47 ± 0.05^ab^	1.52 ± 0.05^b^	1.49 ± 0.05^ab^
PER	1.50 ± 0.13^a^	1.62 ± 0.06^ab^	1.78 ± 0.07^bc^	1.85 ± 0.02^c^	1.76 ± 0.03^bc^
FCR	1.41 ± 0.13^c^	1.32 ± 0.06^bc^	1.21 ± 0.06^ab^	1.14 ± 0.02^a^	1.18 ± 0.03^a^
CF (g cm^−3^)	1.23 ± 0.09	1.24 ± 0.08	1.23 ± 0.06	1.25 ± 0.09	1.25 ± 0.08
VSI (%)	12.88 ± 0.95^a^	12.96 ± 1.23^a^	13.14 ± 1.26^a^	13.65 ± 1.55^ab^	14.29 ± 1.67^b^
HSI (%)	1.13 ± 0.25	1.11 ± 0.10	1.19 ± 0.12	1.23 ± 0.06	1.28 ± 0.16
Survival rate (%)	90.00 ± 1.67^a^	92.22 ± 1.92^a^	91.67 ± 5.00^a^	93.33 ± 2.89^a^	96.67 ± 2.89^b^

*Note:* WG (%) = 100 × (FBW−IBW)/IBW. SGR (% day^−1^) = 100 × (Ln FBW−Ln IBW)/feeding days. PER = WG (g)/protein fed (g). FCR = feed intake (g)/WG (g). CF = fish weight (g) × 100/body length^3^ (cm). VSI = 100 × (viscera weight/whole-body weight). HSI = 100 × (liver weight/whole-body weight). Survival rate (%) = 100 × (final amount of fish)/(initial amount of fish). Values with different superscripts in the same column are significantly different (*p* < 0.05).

Abbreviations: 6FO, 60 g/kg fish oil; 9FO, 90 g/kg fish oil; 12FO, 120 g/kg fish oil; 15FO, 150 g/kg fish oil; 18FO, 180 g/kg fish oil; CF, condition factor; FBW, final body weight (g); FCR, feed conversion ratio; PER, protein efficiency ratio; HSI, hepatosomatic index; IBW, initial body weight (g); SGR, specific growth rate; VSI, viscerosomatic index; WG, weight gain.

**Table 4:** The proximate composition of whole-body fed diets containing varying levels of fish oil (mean ± S.E).

Groups	6FO	9FO	12FO	15FO	18FO
Moisture	78.09 ± 0.05^b^	77.54 ± 0.28^b^	77.50 ± 0.78^b^	76.61 ± 0.34^a^	76.24 ± 0.09^a^
Crude protein	14.54 ± 0.12^b^	14.54 ± 0.20^b^	14.32 ± 0.18^b^	13.96 ± 0.10^a^	14.10 ± 0.28^a^
Crude lipid	4.20 ± 0.23^a^	4.55 ± 0.18^a^	5.16 ± 0.59^a^	6.10 ± 0.33^b^	6.41 ± 0.22^b^
Ash	2.55 ± 0.08	2.58 ± 0.06	2.43 ± 0.09	2.48 ± 0.06	2.45 ± 0.03

*Note:* Values with different superscripts in the same column are significantly different (*p* < 0.05).

Abbreviations: 6FO, 60 g/kg fish oil; 9FO, 90 g/kg fish oil; 12FO, 120 g/kg fish oil; 15FO, 150 g/kg fish oil; 18FO, 180 g/kg fish oil; S.E, standard error of mean.

**Table 5:** Effects of different fish oil levels on the intestinal and liver enzyme activity of Amur grayling (mean ± S.E).

Groups	6FO	9FO	12FO	15FO	18FO
Intestine	—	—	—	—	—
Lipase (U/mgprot)	382.43 ± 40.16^a^	459.94 ± 29.40^b^	501.58 ± 49.36^bc^	547.27 ± 37.19^c^	586.95 ± 48.12^c^
* α*-Amylase (U/mgprot)	0.26 ± 0.20	0.21 ± 0.18	0.25 ± 0.13	0.23 ± 0.23	0.27 ± 0.22
Trypsin (U/mgprot)	4549.29 ± 626.12^a^	5104.94 ± 794.06^a^	4707.77 ± 174.95^a^	6151.61 ± 376.95^b^	7564.48 ± 244.31^c^
SOD (U/mgprot)	19.23 ± 1.43^a^	21.38 ± 2.19^ab^	22.99 ± 2.38^b^	25.43 ± 2.42^c^	26.74 ± 2.11^c^
CAT (U/mgprot)	15.71 ± 3.01^a^	16.40 ± 1.45^a^	17.53 ± 2.11^ab^	22.90 ± 3.85^c^	20.81 ± 3.49^bc^
MDA (nmol/mgprot)	46.27 ± 1.01^b^	42.62 ± 2.17^b^	36.52 ± 1.46^a^	33.91 ± 3.32^a^	51.19 ± 1.93^c^
Liver	—	—	—	—	—
SOD (U/mgprot)	37.50 ± 2.60^a^	38.99 ± 2.25^a^	42.68 ± 2.57^b^	48.44 ± 2.85^c^	44.67 ± 2.79^b^
CAT (U/mgprot)	23.86 ± 2.74^a^	25.21 ± 2.86^a^	28.06 ± 3.90^a^	33.74 ± 3.50^b^	33.21 ± 3.02^b^
MDA (nmol/mgprot)	51.60 ± 2.36^c^	47.53 ± 1.56^c^	40.07 ± 2.78^b^	32.57 ± 2.78^a^	58.56 ± 3.60^d^
LZM (U/mgprot)	1.44 ± 0.12^a^	1.62 ± 0.03^b^	1.83 ± 0.06^c^	2.15 ± 0.03^d^	1.87 ± 0.05^c^
AKP (U/gprot)	1.72 ± 0.16^a^	1.90 ± 0.06^a^	2.25 ± 0.10^b^	2.54 ± 0.07^c^	1.78 ± 0.08^a^
ACP (U/gprot)	4.85 ± 0.45^a^	5.15 ± 0.40^a^	6.09 ± 0.29^b^	7.98 ± 0.32^c^	4.82 ± 0.23^a^

*Note:* Values with different superscripts in the same column are significantly different (*p* < 0.05).

Abbreviations: 6FO, 60 g/kg fish oil; 9FO, 90 g/kg fish oil; 12FO, 120 g/kg fish oil; 15FO, 150 g/kg fish oil; 18FO, 180 g/kg fish oil; ACP, acid phosphatase; AKP, alkaline phosphatase; CAT, catalase; LZM, lysozyme; MDA, malondialdehyde; SOD, superoxide dismutase.

## Data Availability

The raw sequences of the 16S rRNA gene can be found in the NCBI Sequence Read Archive (SRA; accession no. PRJNA1043406).

## Supporting Information

Supporting Information 1 Information on the primer sequences.

Supporting Information 2 Rarefaction curve analysis of intestinal microbial diversity.

Supporting Information 3 Alpha diversity indices of the intestinal microbial community.

Supporting Information 4 Principal Coordinate Analysis (PCoA) of the intestinal microbial communities at the genus level.

Supporting Information 5 Venn diagram analysis of shared and unique OTUs between groups at the genus level.
