# Supplementary material for: Effects of Dietary Fish Oil Levels on Growth Performance, Lipid Metabolism, Hepatic Health, Nonspecific Immune Response, and Intestinal Microbial Community of Juvenile Amur Grayling (Thymallus grubii)
Source: Aquac Nutr. 2024 Nov 21;2024:8587410. doi: 10.1155/anu/8587410 (PMC11606657; doi:10.1155/anu/8587410)
Supplement: Supporting Information 1 — Information on the primer sequences. [file 8587410.f1.docx]

Table S1 Primer sequences and slope values

| Gene | Slope value | Primer sequence (5’-3’) | |
| --- | --- | --- | --- |
|  |  | Forward | Reverse |
| PPAR-γ | 0.0669 | GAGGGAGAGGCAACGTCAAA | TCCCCGTCATCTCTCAGGTT |
| FAS | 0.0950 | ACCTCTTCCTCTTCGACGGA | TTGACACGGGCCTTCAGATC |
| CPT1A | 0.0368 | TACGCTTCAAGAACGGGGTC | ACTGATGGGTATGTGGCTGC |
| TGF-β | 0.0328 | AGCAGGGCAACTCTGATGAC | CTGGTGAATGATGGCCAGGT |
| NF-κB | 0.0285 | AACGACCTCACAGCACAGTT | TGATAGTGCTGCCCCCTTTG |
| IL-8 | 0.0308 | GACCGAGAGCAAACGCATTG | TGACCCTCTTGACCCAAGGA |
| β-actin | 0.0794 | GCTCTGCCCCACGCCATCCT | CGGTGCCCATCTCCTGCTCAAAG |
