# Supplementary material for: Effects of Dietary Fish Oil Levels on Growth Performance, Lipid Metabolism, Hepatic Health, Nonspecific Immune Response, and Intestinal Microbial Community of Juvenile Amur Grayling (Thymallus grubii)
Source: Aquac Nutr. 2024 Nov 21;2024:8587410. doi: 10.1155/anu/8587410 (PMC11606657; doi:10.1155/anu/8587410)
Supplement: Supporting Information 2 — Rarefaction curve analysis of intestinal microbial diversity. [file 8587410.f2.docx]

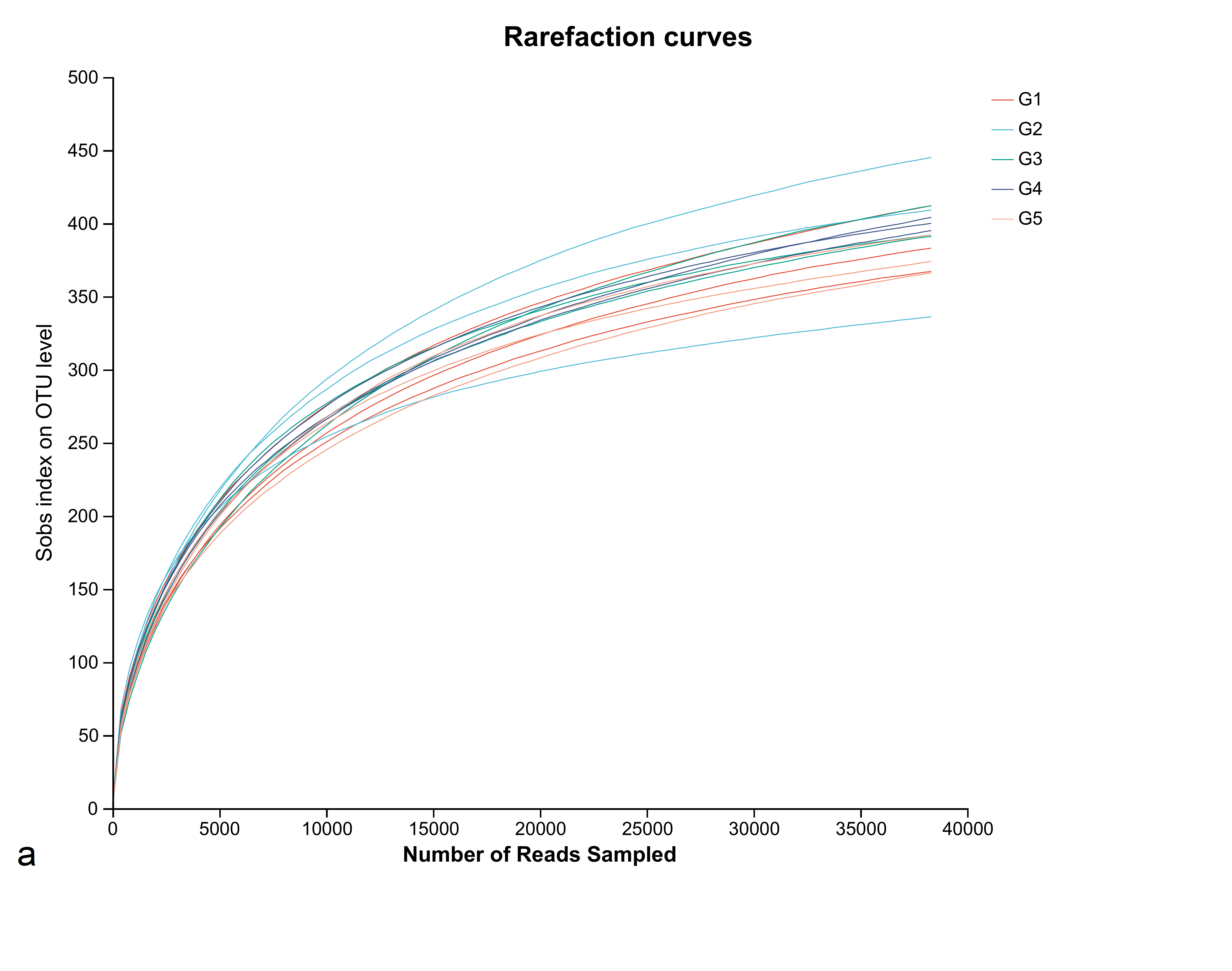

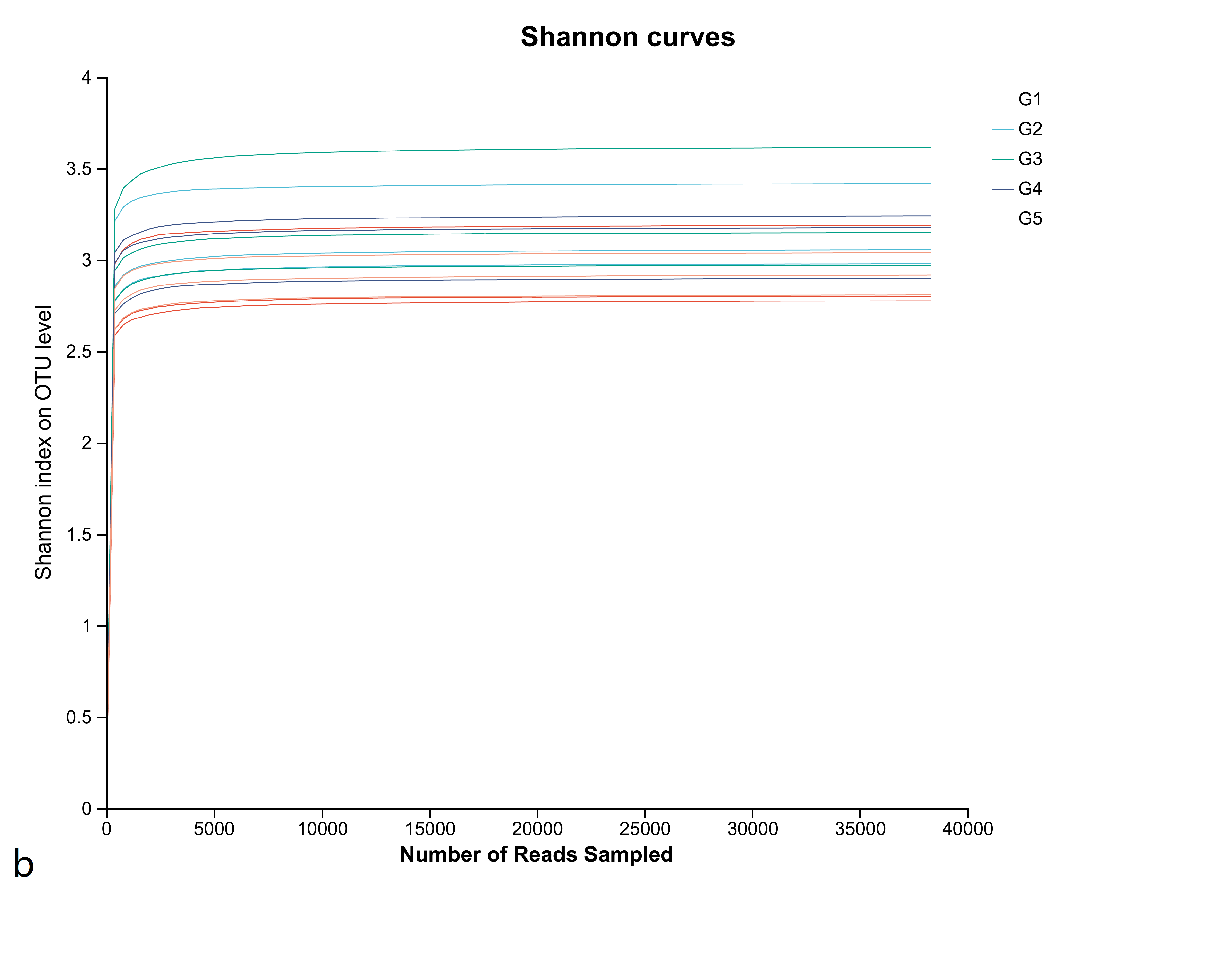
Figure S1 Rarefaction curve analysis of intestinal microbial diversity using the Sobs index (a) and the Shannon index (b) to assess operational taxonomic units (OTUs).
