# Supplementary material for: Effects of Dietary Fish Oil Levels on Growth Performance, Lipid Metabolism, Hepatic Health, Nonspecific Immune Response, and Intestinal Microbial Community of Juvenile Amur Grayling (Thymallus grubii)
Source: Aquac Nutr. 2024 Nov 21;2024:8587410. doi: 10.1155/anu/8587410 (PMC11606657; doi:10.1155/anu/8587410)
Supplement: Supporting Information 3 — Alpha diversity indices of the intestinal microbial community. [file 8587410.f3.docx]

Table S2 Alpha diversity indices of the intestinal microbial community of juvenile Amur grayling fed diets containing graded fish oil levels （Mean ± S.E.）

| Item | L103.5 | L128.9 | L157.3 | L185.3 | L221.6 |
| --- | --- | --- | --- | --- | --- |
| ace | 391.59±32.00 | 391.92±54.70 | 414.95±55.58 | 403.40±13.37 | 375.37±10.05 |
| chao | 392.61±31.14 | 408.52±48.74 | 423.86±32.86 | 413.51±22.64 | 394.36±9.00 |
| shannon | 2.89±0.22 | 3.09±0.21 | 3.04±0.10 | 3.09±0.09 | 2.86±0.12 |
| simpson | 0.14±0.03 | 0.14±0.02 | 0.13±0.02 | 0.11±0.02 | 0.14±0.02 |
| Shannon/Simpson | 21.44±6.78 | 22.55±2.69 | 24.50±4.53 | 29.54±5.34 | 21.40±3.56 |
| coverage | 99.82±0.03 | 99.83±0.04 | 99.82±0.03 | 99.81±0.02 | 99.80 ±0.02 |
