# Supplementary material for: Effects of Dietary Fish Oil Levels on Growth Performance, Lipid Metabolism, Hepatic Health, Nonspecific Immune Response, and Intestinal Microbial Community of Juvenile Amur Grayling (Thymallus grubii)
Source: Aquac Nutr. 2024 Nov 21;2024:8587410. doi: 10.1155/anu/8587410 (PMC11606657; doi:10.1155/anu/8587410)
Supplement: Supporting Information 4 — Principal Coordinate Analysis (PCoA) of the intestinal microbial communities at the genus level. [file 8587410.f4.docx]

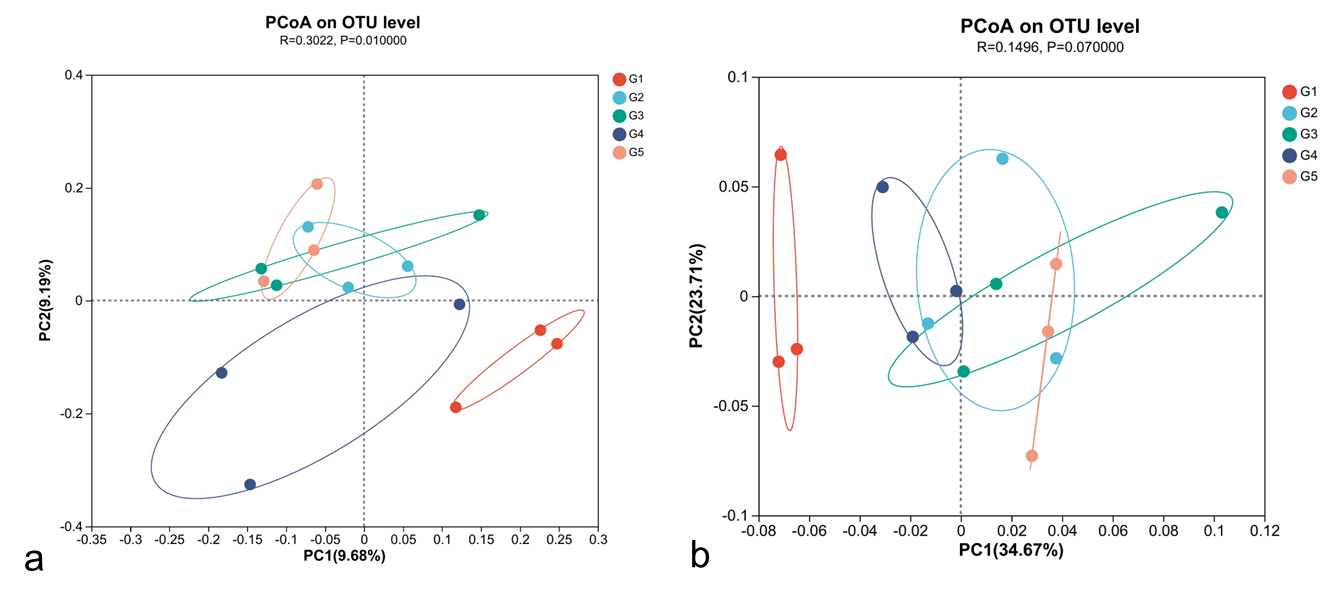


Figure S2 Principal co-ordinate Analysis (PCoA) of the intestinal microbial communities at the genus level based on binary Jaccard (a) and weighted Unifrac (b) metrics. G1: 6FO, G2: 9FO, G3: 12FO, G4: 15FO and G5: 18FO.
